# An All-in-One Dual Band Blade Antenna for ADS-B and 5G Communications in UAV Assisted Wireless Networks

**DOI:** 10.3390/s21175734

**Published:** 2021-08-25

**Authors:** Maximilian James Arpaio, Giacomo Paolini, Franco Fuschini, Alessandra Costanzo, Diego Masotti

**Affiliations:** Department of Electrical, Electronic and Information Engineering (DEI) “G. Marconi”, Alma Mater Studiorum University of Bologna, 40136 Bologna, Italy; giacomo.paolini4@unibo.it (G.P.); franco.fuschini@unibo.it (F.F.); alessandra.costanzo@unibo.it (A.C.); diego.masotti@unibo.it (D.M.)

**Keywords:** 5G, automatic dependent surveillance-broadcast, blade antenna, detect-and-avoid, unmanned air vehicle

## Abstract

This paper is aimed at the characterization and manufacturing of an SMA coaxial fed compact blade antenna with dual frequency characteristics for broadband applications on board of Unmanned Air Vehicles (UAVs). This antenna is linearly polarized, and it combines the benefits of Automatic Dependent Surveillance-Broadcast (ADS-B) and 5th Generation (5G) communications in one single element, covering both the 1.030–1.090 GHz and the 3.4–3.8 GHz bands thanks to a bent side and a ‘C’ shaped slot within the radiation element. Starting from the simulation outcomes on an ideal ground plane, the results are here extended to a bent ground plane and on two UAV commercial CAD models. Details of manufacturing of the antenna in both aluminium and FR-4 substrate materials are presented. The comparison between measurements and simulations is discussed in terms of return loss, bandwidth, gain, and radiation pattern. Results show an antenna with a low profile and a simple structure that can be employed in various wideband communication systems, suiting future UAV assisted 5G networks while being perfectly compliant with forthcoming ADS-B based Detect-And-Avoid (DAA) technologies in Unmanned Aerial Traffic Management (UTM).

## 1. Introduction

A few decades ago, Unmanned Aerial Vehicles (UAV), also known as drones, were confined to science fiction or notions of the future. Today, unmanned Aircraft Systems are rapidly becoming part of our everyday life and they have found a widespread use and for a wide range of applications [1,2,3,4,5,6,7,8], especially in the form of small quadcopters and octocopters, owing their success to their flexibility and potential cost efficiency in comparison with conventional aircrafts or ground-based infrastructures. Although originally conceived only for military purposes, nowadays drones have seen rapid growth and advancements and finally made a break to consumer electronics, quickly increasing in numbers and complexity. Examples of missions currently accomplished by UAVs include for instance infrastructure inspections, real estate imagery, aerial photography and video, inventory control, news gathering, aerial sensing for agriculture, movie and television production, perimeter surveillance, facility security, and many others. Among the different drivers, a combination of maturing and sophisticated flight technologies, small-size, high-performance sensors, and the widespread availability of low-cost platforms has driven an explosive growth in the applications of UAVs for commercial and humanitarian purposes [9]. UAV has gained an immense popularity also among amateurs and academic researchers as suitable enablers for many emerging technologies, trials, and applications, a trend likely to continue in the next future. In [10], it is estimated that the European drone market will represent EUR 10 billion annually by 2035 and over EUR 15 billion annually by 2050. Because of this growth and market trend, the projected expansion of UAV operations worldwide has fostered the urgent need to evaluate how these aircraft will “see and be seen” by other aircraft operating inside the same airspace. In the next decade, the growing number of operations is expected to drive a large increase in air traffic volume at low altitudes and for this reason new technologies and procedures will be required to ensure safe operations also at such low altitudes.

Surveillance and communication between these UAV and other aircraft is, therefore, critically important to allow operations. Most of the effort to safely integrate manned and unmanned aircraft within the same airspace focuses on the Detect-And-Avoid (DAA) technology.

**Figure 1:**
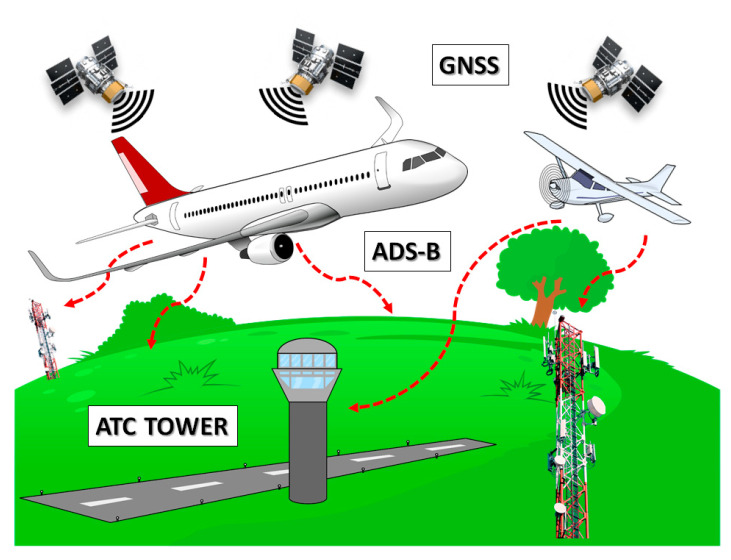
ADS-B layout, showing aircraft receiving GNSS signal and transmitting ADS-B signals (red dashed arrows) to ATC and receiving ground stations on high masts.

In fact, just as manned pilots must “see and avoid” other aircraft, drones need to be able to avoid colliding with other aircraft, and that must be as automated as possible. DAA approaches are generally divided between *cooperative* (e.g., TCAS, Mode-C/S, …) and *non-cooperative* (e.g., visible, infrared, and radar). Among the different cooperative proposals available in the market or under development, the most promising one relies on a datalink that is already in use and compatible with aviation, such as the Automatic Dependent Surveillance-Broadcast (ADS-B) service [11]. Driven by the need to implementing a more accurate way of tracking and surveilling aircraft positions, ADS-B uses the Global Navigation Satellite System (GNSS) to identify aircraft locations and radio signals in the 1090 MHz frequency band to share that data in real time (Figure 1).

ADS-B has been already proven to be effective and efficient in aviation surveillance, being it widely used in commercial aviation for years in the United States, Canada, Australia, India, and Europe, and becoming an increasingly important part of the aviation safety ecosystem worldwide, as it can be further extended to UAVs. In this regard, there has been a lot of movement in 2020 in leveraging ADS-B as a key DAA solution. As an example, DJI, the largest consumer drone manufacturer, recently announced its intention to include ADS-B receivers in all its new models for consumer drones weighing over 250 g starting in 2020 [12], dramatically reducing risk and improving situational awareness since drones are very hard to see (and avoid) from the airplane cockpit. Not to mention that a drone quickly goes from a role of a huge asset to one of liability in the blink of an eye if GPS is lost. Remote pilots must always yield right of way to any manned aircraft, and that is not always easy when trees, buildings, and other obstacles can obscure an approaching aircraft. Eyes and ears alone may not always leave a remote pilot enough time to get out of the way of an approaching aircraft.

Just like in the cockpit, ADS-B on drones gives the pilot precious minutes to detect, decide, and act, especially during operations beyond Visual Line of Sight (VLOS), something the market and the research community are looking forward to.

**Figure 2:**
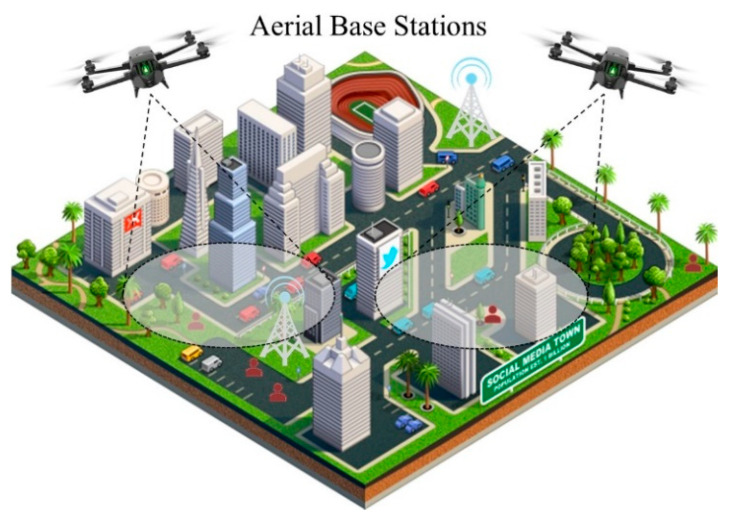
Aerial base stations providing 5G services to users at ground level in an urban environment.

At the same time, Unmanned Aerial Base Stations (UABSs), i.e., base stations carried by UAVs, is one of the most promising means to offer coverage and capacity in 5G applications to those users that are not being served by terrestrial base stations, with a special focus on the extension of coverage and capacity of mobile radio networks [13]. In cellular communications, it is well-known that a wireless connection to the public cellular network is established through the local cell towers. Unfortunately, during a natural disaster or a simple out-of-service, these towers may lose their functionality, and this leads to a subsequent loss of communication in the area. In such situations, constant coverage and communication are vital for public safety, especially when there is need for coordination of different activities. In this case, UAVs can establish instant connectivity by forming ad-hoc networks to replace the malfunctioning tower in the cellular network. Once set up, the UAVs can act as mobile base stations and start routing traffic to and from the cell tower (backbone) while serving users at ground level with 5G or any next generation services (Figure 2). Similar techniques can be used either in remote or urban areas that either lack cellular coverage or need to increase their capacity due to congested networks (and unsatisfied users).

**Figure 3:**
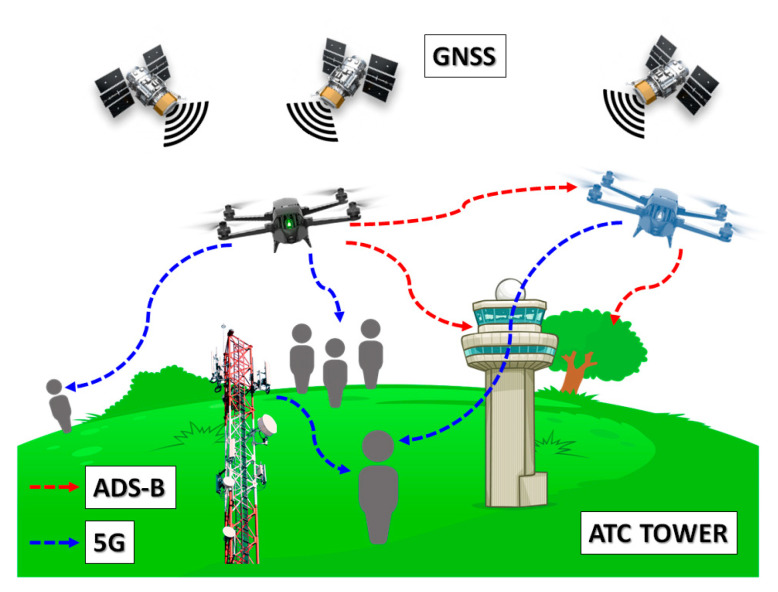
The envisaged UAVs applications, operating 5G services (blue dashed arrow) for users at ground level while improving DAA awareness via ADS-B (red dashed arrow).

The purpose of this work is, thus, to introduce a dual-mode antenna with suitable relative bandwidth to support UAV applications in 5G networks while granting DAA over ADS-B link (Figure 3). Similarly to [14], this paper focuses the research at compact size, light weight, and multi-frequency band antenna. Specifically, the novelty in the proposed dual band solution has been envisaged and preliminarily investigated for the first time in [15], and it is here further validated and supported by means of additional simulations and thorough comparison with experimental results.

After a brief introduction and motivations about the ADS-B link technology and UABSs philosophy for 5G networks in Section 1, Section 2 describes the antenna solution, focusing on both ADS-B and 5G frequency bands while showing the way the two can co-exist. Section 3 deals with the effect of a curvature radius on the ideal ground plane, secondly accounting for a real UAV fuselage body, while Section 4 introduces the manufacturing of the antenna prototypes and their experimental results. Section 5 draws the main conclusions.

## 2. Dual Band Blade Antenna Solution

Blade antennas are classified as planar monopole resonators and are often considered an effective solution in airborne applications because of their low wind resistance, aerodynamic features, light weight, simplicity of fabrication, and wide bandwidth. Limitation on the total height of antenna and ground plane size in UAV applications require specific design caution, while a radome may be sometimes helpful to protect the radiator element when the antenna on UAV is exposed to climate variations.

A blade antenna with an oblique side, a ‘C’ shaped slot and SMA connector has been introduced for the first time in [15] to address these issues. In accordance with [16], the oblique side on the blade allows the antenna to achieve slightly wider impedance bandwidth. Due to constraints on the total height of the antenna in airborne applications, the height of the blade is limited by design to less than 65 mm (λ/4 at 1.1 GHz). In accordance with previous works [17,18,19], adding a slot—of a specific shape—provides another resonating frequency, in this case at around 3.5 GHz, since the resonating cavity has a length of less than 20 mm (λ/4 at 3.5 GHz).

The main steps underlying the antenna design on ideal ground plane are shortly resumed herein from [15], whereas comparisons between simulation/measurements and investigations on antenna performance when placed on real UAVs frames are addressed in the following sections.

**Figure 4:**
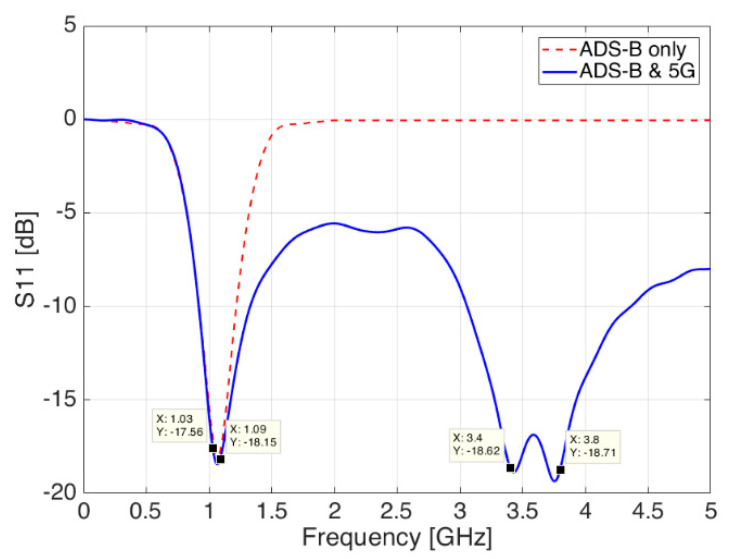
Reflection coefficient S_11_ of simulated antenna; ADS-B only (red dashed line) and ADS-B and 5G (blue continuous line)—Aluminium solution.

Starting from basic antenna theory equations [20,21], the antenna model was first created in CST Microwave Studio suite and simulated over a nearly infinite ground plane in order to get resonance in the lowest frequency band, which must be centered at 1.090 GHz according to ICAO specs [22] (red, dashed line in Figure 4). It should be pointed out from Figure 4 the availability of the 1.030 GHz frequency, in case of possible implementation of interrogation needs for Mode S, Mode A/C, TCAS or even Airborne Collision Avoidance System (ACAS) [23] on board of UAVs.

The second resonating frequency, needed for 5G communications, has been then added by carving a resonant cavity—also called slot—onto the blade surface. Slots can be easily concealed inside metallic objects, or they can also be carved on the surface, giving in return good performances in terms of bandwidth and gain, while being easy to manufacture. After a new optimization phase, the reflection coefficient shows still a good resonance at 1.090 GHz and a new one at 3.5 GHz (blue continuous line in Figure 4).

**Figure 5:**
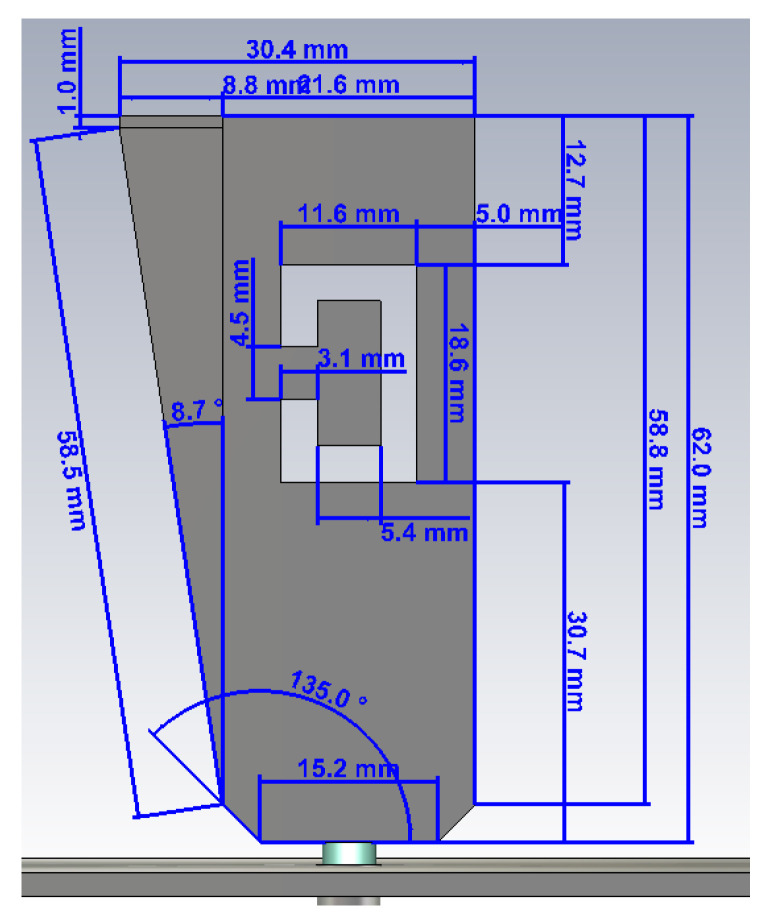
Final dimensions of the blade antenna in dual frequency configuration (XZ side view).

Radiation diagrams on H-V planes for this final design are described in Section 4 together with their experimental results. The proposed structure, including final design and dimensions [15], is shown in Figure 5.

## 3. Antenna Placement on Real UAV Frames

The next analysis step is to investigate how the designed antenna behaves when placed on the body of a generic aircraft. This is particularly true in case of blade antennas, as the ground plane plays an important role in both the matching and the radiation diagram. On one hand, the metallic fuselage of an aircraft easily replaces and behaves as a reasonable ground plane; on the other hand, it might not be a perfectly flat surface of infinite length, but it is bent and limited in size, especially in some directions, and as such it can affect the performances of the antenna.

Performances in terms of return loss have been investigated under the ideal case of a set of three uniform fuselage curvature radius R, both in the X-axis and in the Y-axis directions. The radius R was chosen with the following three bending values R = 50, 275, and 500 mm. A third and final evaluation has been carried out in case of real 3D drone models used as a ground plane.

### 3.1. Variation of the Bending Radius along X-Axis

**Figure 6:**
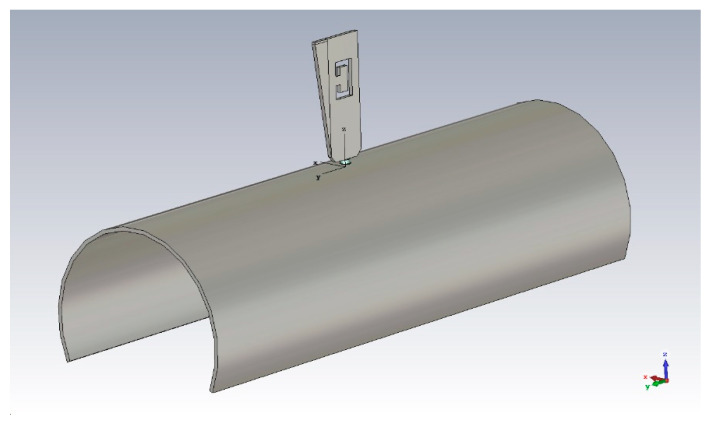
Bent ground plane, ‘X’ direction (R = 50 mm, example)—perspective view.

**Figure 7:**
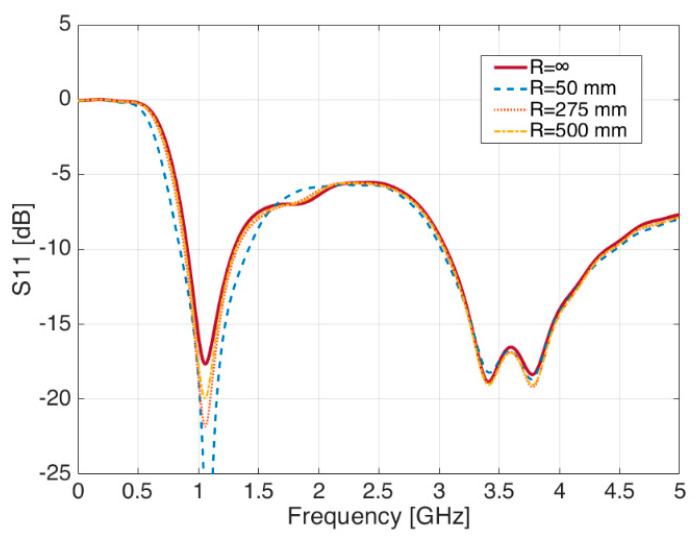
Reflection coefficient S_11_ for different values of ‘R’—Bending in the ‘X’ direction.

In case the bending radius occurs in the ‘X’ direction (Figure 6), a parametric sweep simulation shows changes in the S_11_ plot, with general improvements in the matching conditions of the 1.090 GHz frequency band, as it can be seen in Figure 7. Minor changes—if no change at all—are observed on the contrary in the S-band at 3.5 GHz.

By means of the same parametric sweep on the bending radius R, it is possible to see meaningless variations of the radiation diagram in the XY-, XZ-, and YZ-planes for both frequencies, meaning the radiation diagrams are definitely not sensitive to the variations of the ground plane.

### 3.2. Variation of the Bending Radius along Y-Axis

**Figure 8:**
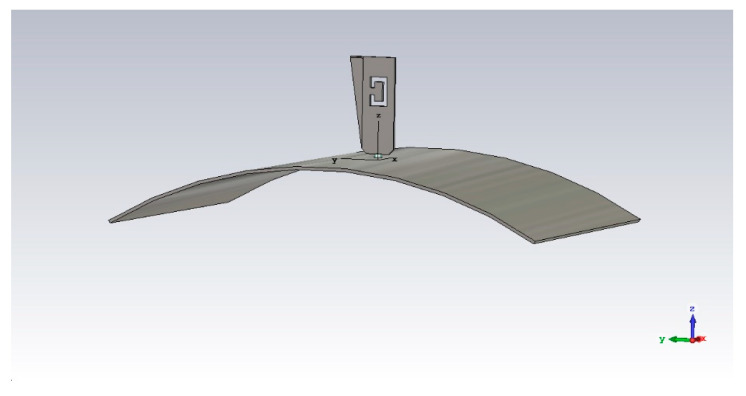
Bent ground plane, ‘Y’ direction (R = 275 mm, example)—perspective view.

In case the ground plane is bent in the ‘Y’ direction (Figure 8), S_11_ shows a tiny shift in frequency in the 1.090 GHz band, while the S-Band shows a general improvement in the matching.

**Figure 9:**
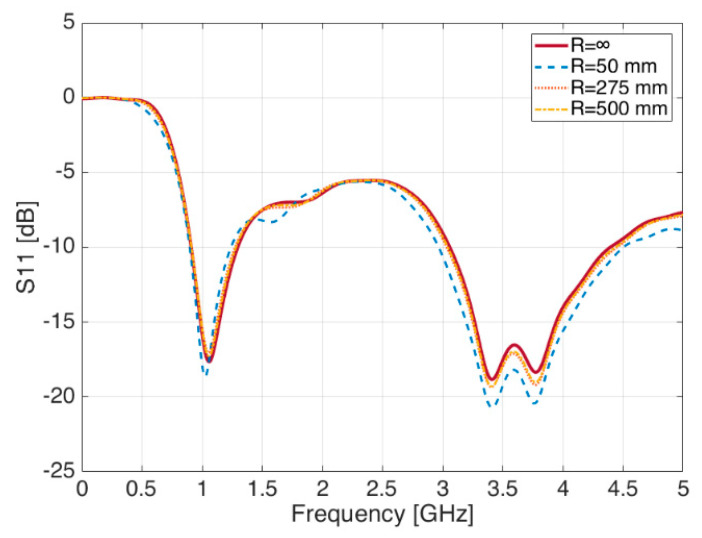
Reflection coefficient S_11_ for different values of ‘R’—Bending in the ‘Y’ direction.

The following Figure 9 summarizes the outcomes of the simulation runs, under different radius sweeps.

The slight sensitivity of the reflection coefficient with regard to the curvature of the ground plane could be somehow related to the surface portion and distance of the ground plane area available below the blade antenna, which probably modifies the current distribution and the interactions between the different frequency bands.

Regarding the effects on the radiation diagrams, little variations in the XY-, XZ-, and YZ-planes for both frequencies, are seen but not significant enough to report remarkable notes among the outcomes of the paper.

From this analysis, it is possible to state that, as long as the ground plane size and the bending radius that shapes the fuselage frame are within acceptable values, i.e., the drone fuselage curvature is not too sharp, the proposed blade antenna shows negligible impact on its radiation and matching performances, which proves the design introduced in Section 2 to be robust enough against those variations.

### 3.3. Fuselage of Commercial Drones

When dealing with UAVs, it is important to understand how the previous results change when the blade antenna is placed on real UAVs, which can be covered in composite or plastic materials, with the metallic parts laying underneath. Similar evaluations have been already carried out for commercial aircraft [24,25,26] where the fuselage is many wavelengths bigger than the antenna and the operating frequency, but as far as the authors are concerned, literature currently lacks similar studies for UAV bodies although a preliminary assessment has been introduced in [27].

**Figure 10:**
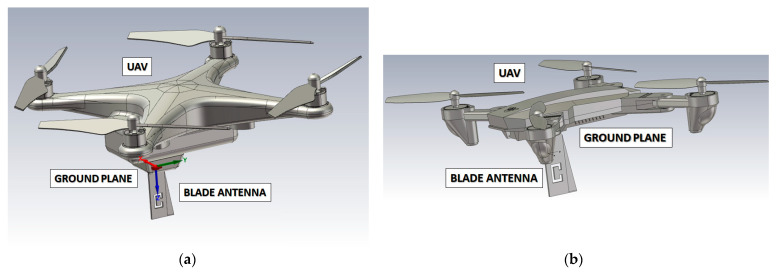
DJI Phantom 3 (**a**) and Mavic Pro (**b**) drone models with the aluminium blade antenna placed below the body.

In this research activity, it was used a SolidWorks model (STL file) to simulate two major UAVs from DJI, below which the antenna was placed: the Mavic Pro and the Phantom 3 [28]. To facilitate transmission towards the ground, the antenna is assumed to be mounted upside-down below the UAV body, using the airframe as a candidate ground plane (Figure 10a,b). Please note that landing skids—made of light plastic—were removed from the 3D model to simplify it and to reduce the simulation time.

The two drones have been modelled assuming metallic parts for the motors, together with carbon fiber for the four propellers. The body on the contrary is assumed to be made for simplicity of simulation of tough Polylactic Acid (PLA), a well-known plastic polyester composite for drones (ε_r_ = 2.62 and tanδ = 0.014 at 1 GHz), with a metallic core beneath to account for both the electronic circuitry and the thin frame.

**Figure 11:**
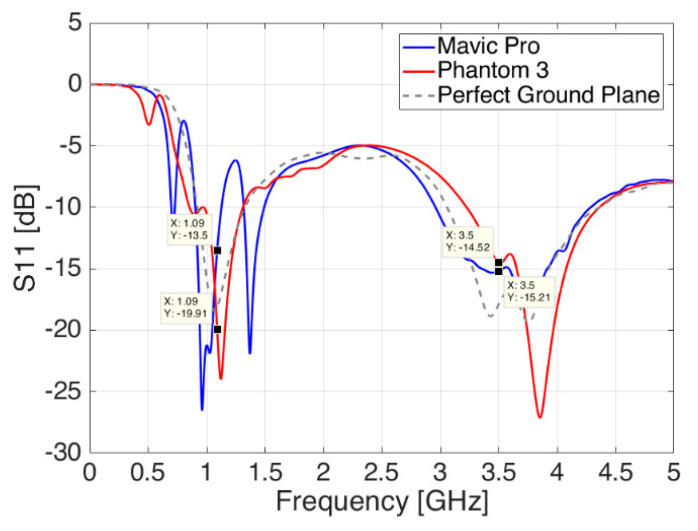
Reflection coefficient S_11_ of the blade antenna—ideal ground plane case (dashed grey line) compared to the antenna placement on DJI Phantom 3 (red line) and DJI Mavic Pro (blue line).

Similar to previous sections, the following picture shows the variations of the antenna reflection coefficient S_11_ for both drone cases. As it can be seen in Figure 11, placing the blade antenna below an actual drone body introduces a change in the antenna reflection coefficient, which leads to another—different—response than the one seen in the previous two subsections.

The Mavic Pro—of more compact size and with a relatively bigger ground plane—adds new ripples and a backward shift of 100 MHz at lower frequencies together with impaired matching values, around 3 dB higher, at both frequencies namely for ADS-B and 5G communications. The Phantom 3—bigger in size but with a smaller available ground plane—adds a forward shift of around 50 MHz at both frequencies, thus, moving the antenna matching away from its optimum values. Nevertheless, in both drone body cases, the changes are still within acceptable values, and they could be addressed during actual installation phase by means of local antenna adaptations in relative position and distance from the ground plane in order to minimize the effects of the drone rotors and the landing gear skids next to it, especially at the higher 5G frequencies.

**Figure 12:**
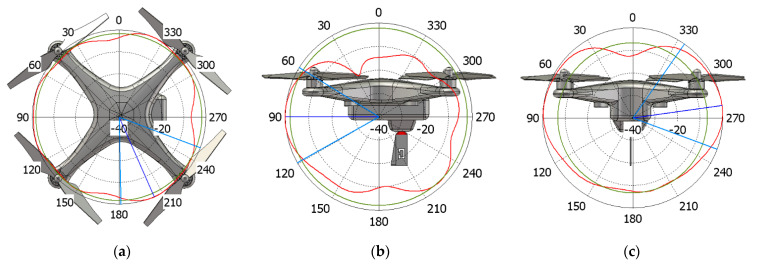
Normalized gain pattern (red line) for DJI Phantom 3 model at 1.09 GHz, (**a**) XY plane, (**b**) YZ plane, and (**c**) XZ plane—CST Simulation Results in dB.

**Figure 13:**
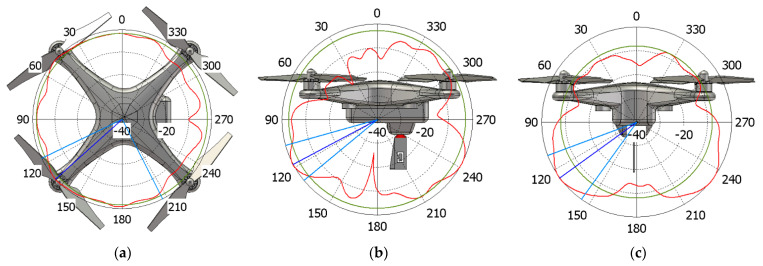
Normalized gain pattern (red line) for DJI Phantom 3 model at 3.5 GHz, (**a**) XY plane, (**b**) YZ plane and (**c**) XZ plane—CST Simulation Results in dB.

With reference to the radiation diagrams, the body of the UAV interacts in a different and more complex way when compared to the ideal ones acquainted in [15]. By focusing on the DJI Phantom 3 model as an example, it is possible to evaluate field cuts at both 1.09 and 3.5 GHz. From the two sets of simulations shown in Figure 12 and Figure 13 in logarithm scale (dB), it can be seen from the red line that the blade antenna provides fairly seamless 360° coverage around the drone in the XY-plane. A special imprint is seen on the contrary on the YZ and XZ-plane, thus, enhancing the directions towards the ground when installed upside down, as targeted in the design phase of Section 2. The green circles show the side/back lobe levels within the same radiation patterns. Although not reported, similar results are seen also in case of the Mavic Pro drone.

## 4. Manufacturing and Experimental Results

The first antenna prototype was fabricated using an aluminium sheet with a thickness of 4 mm and cut using laser technology under machine-controlled tolerances. The material was then drilled and the SMA connector was fastened on the ground plate by means of small screws while its central pin was connected on the blade antenna, passing through the hole in the middle of the ground plane sheet. Aluminium was chosen in the first place as a matter of opportunity and assuming that an all-metallic solution would have given more robustness to the antenna.

**Figure 14:**
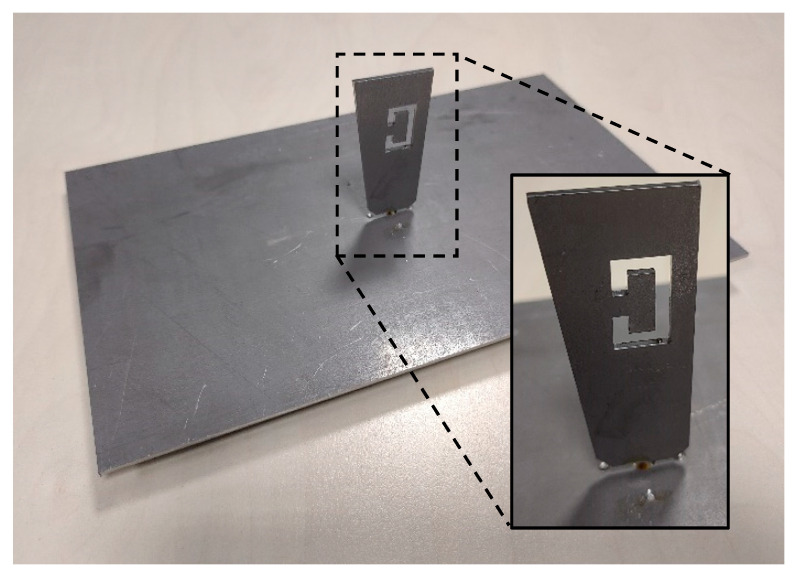
Manufactured blade antenna on a ground plane, including a pop-up box with detailed view of the ‘C’ shaped slot.

A mechanical template was used to keep as far as practical by design the same installation distance d = 2 mm between the blade antenna and the ground plane. The process was relatively easy and fast, and it took only a few hours to be completed with acceptable results (Figure 14). The final antenna weighs around 30 g.

The main antenna parameters were measured experimentally inside a Compact Antenna Test Range (CATR) anechoic chamber from Orbit (now MGV [29]) controlled by MIDAS software processing kit [30].

**Figure 15:**
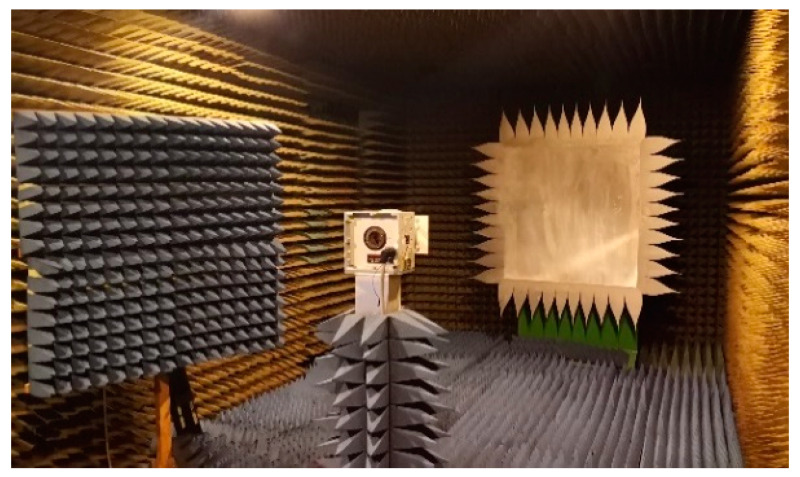
CATR anechoic chamber with reflector and serrations, Rx antenna on rotating mast and Tx antenna (behind absorbing wall cones).

The CATR anechoic chamber has an average size of 9 m × 3.5 m × 3 m and it creates a sufficient section of plane-waves, known as “Quiet Zone”, simply using one reflector to reduce the chamber size (Figure 15). This allows measurements from 12 GHz down to 900 MHz.

**Figure 16:**
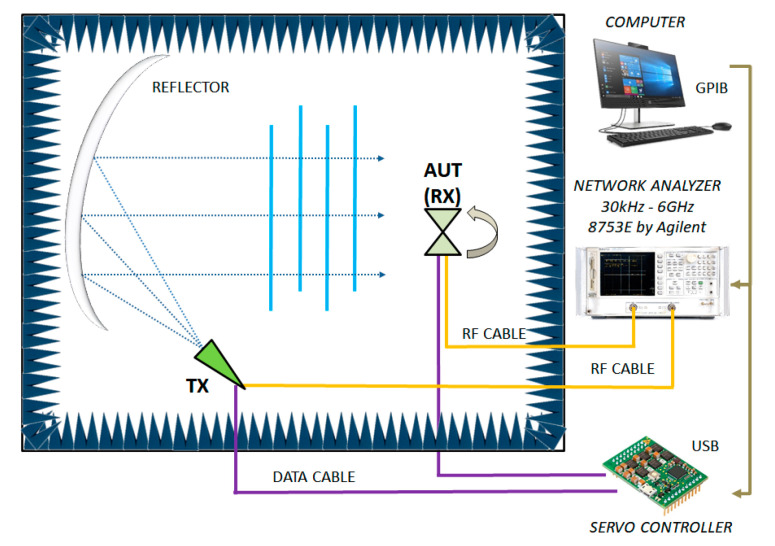
Layout of the anechoic chamber used for the antenna measurement, including interconnection details.

The following picture (Figure 16) shows a functional sketch of the same CATR anechoic chamber used for the blade antenna measurements, including details of connections and test instruments.

**Figure 17:**
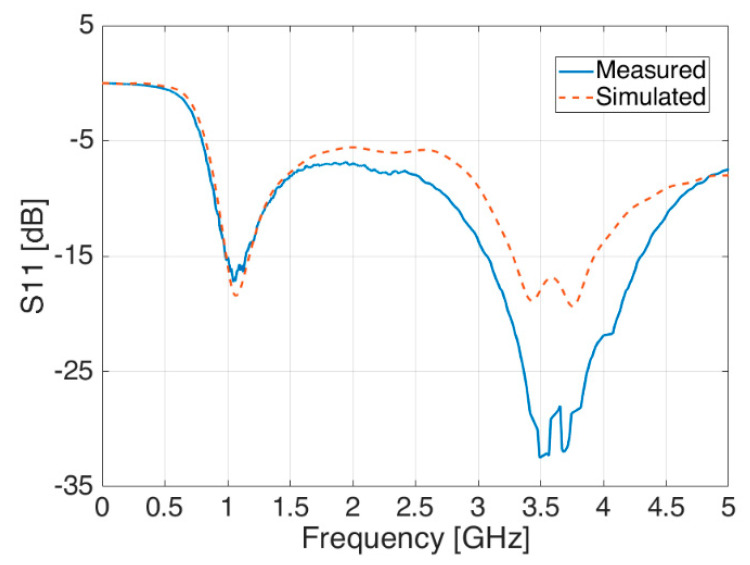
Reflection coefficient S_11_—measured (blue line) and simulated (red dashed line).

**Figure 18:**
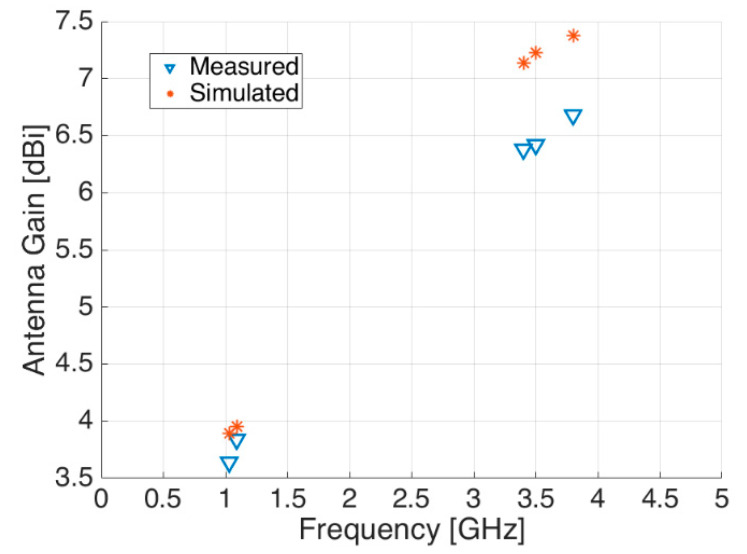
Antenna gain—measured (blue triangle) and simulated (red asterisk).

By looking at other antenna parameters, the same agreement between measurements and simulations is also demonstrated in Figure 17 and Figure 18, where the reflection coefficient S_11_ and the antenna gain are respectively shown.

**Figure 19:**
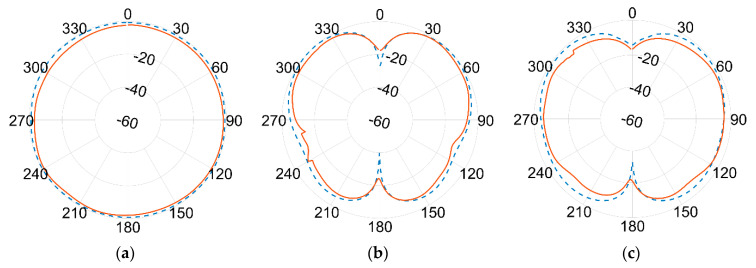
Normalized gain pattern of the aluminium prototype at 1.09 GHz, (**a**) XY plane, (**b**) XZ plane, and (**c**) YZ plane—Simulated (blue dashed line) and Measured (red line) data in dB.

**Figure 20:**
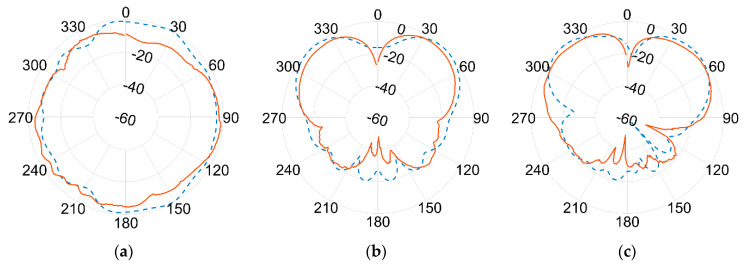
Normalized gain pattern of the aluminium prototype at 3.5 GHz, (**a**) XY plane, (**b**) XZ plane, and (**c**) YZ plane—Simulated (blue dashed line) and Measured (red line) data in dB.

Results of the measured gain radiation diagrams are shown in logarithm scale (dB) in the next page, in Figure 19 and Figure 20, respectively, for 1.09 GHz and 3.5 GHz frequencies and for different crossing planes.

An excellent agreement between measurements (red continuous line) and simulations (blue dashed line) is achieved at the lower frequency. The agreement is still remarkably good at the higher frequency band, although minor discrepancies can be seen. This seems to be more evident in the 180° direction, where the effect of the ground plane and the antenna bracket are assumed to become more relevant at 3.5 GHz than at 1.09 GHz due to the comparable sizes in terms of wavelength λ.

Again, the best fit between simulations and measurements is found at the lower frequencies, i.e., the one used by ADS-B for DAA. Although some differences turn up at higher frequencies, the overall agreement is fairly good all over the two frequencies of interest. According to actual measurements, the antenna proved to be linearly polarized, with vertical polarization on both bands.

### FR-4 Prototype of Blade Antenna

To take advantage of the flexibility of the antenna design shown in Section 2 and to enrich the experimental evaluation towards cheaper, lighter, and more compact materials, the experimental manufacturing was extended to FR-4 substrate material (1.53 mm-thick, with ε_r_ = 4.5 and tanδ = 0.025 at 2 GHz) already available at our University workshop, which provides a second solution, generally more suitable for UAVs. As a matter of fact, thanks to its reduced weight, compared to aluminium, FR-4 should better address battery lifetime of drones and reduce its payload weight.

**Figure 21:**
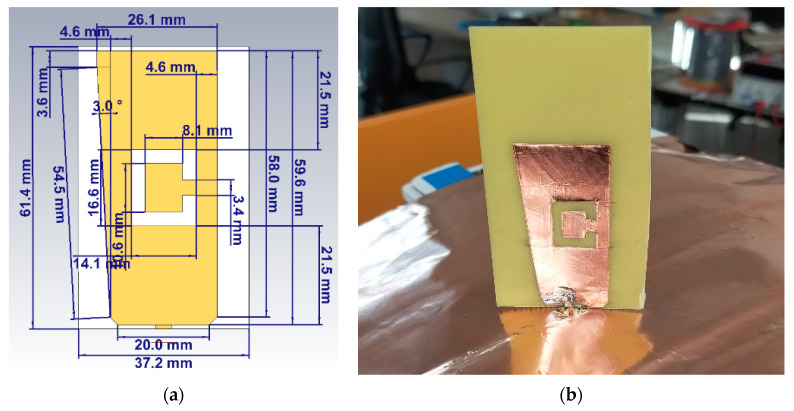
Side view of the blade antenna in FR-4 material, (**a**) 3D model with final dimensions (XZ side view) and (**b**) actual prototype (XZ side view). It is possible to see both the blade-shaped monopole on FR-4 substrate and the ground plane sheet made of thin copper.

The size and length of the new antenna have been optimised during multiple full-wave simulations to consider the new material properties. Simulation results returned on the average 8–10% reduction in size (scaling) of the whole assembly, from the blade monopole height to the ‘C’ shaped slot (Figure 21a).

**Figure 22:**
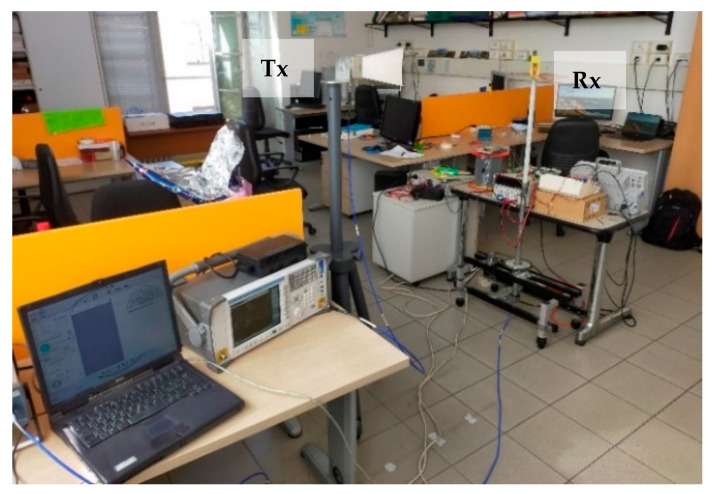
Experimental measurement setup at DEI laboratory.

Thanks to the greater ductility of the adhesive copper sheet (31.75 μm-thick), manufacturing of the single side did not require laser cutting and the antenna has been directly arranged at the University of Bologna (Figure 21b), where an experimental set-up was also deployed in a real office scenario (Figure 22) in order to retrieve the main antenna parameters. The final FR-4 antenna weighs around 7 g, which is 1/3rd of the aluminium case.

**Figure 23:**
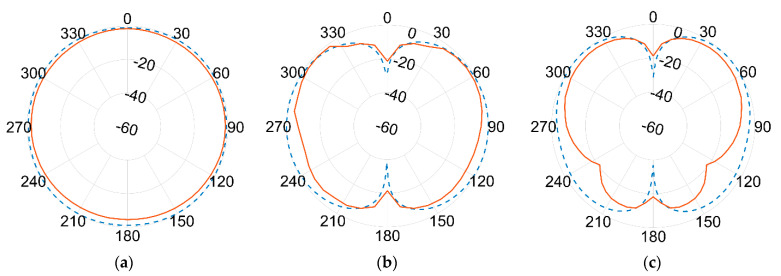
Normalized gain pattern of the FR-4 prototype at 1.09 GHz, (**a**) XY plane, (**b**) XZ plane, and (**c**) YZ plane—Simulated (blue dashed line) and Measured (red line) data in dB.

**Figure 24:**
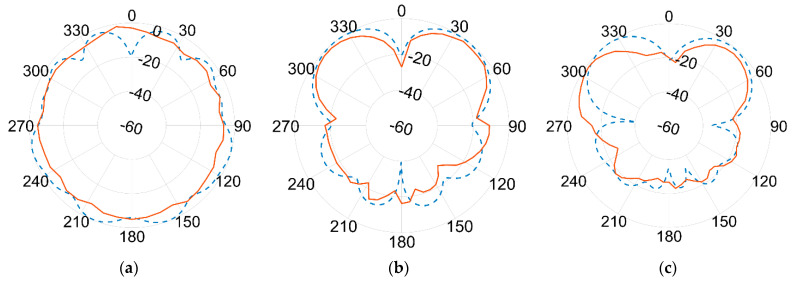
Normalized gain pattern of the FR-4 prototype at 3.5 GHz, (**a**) XY plane, (**b**) XZ plane, and (**c**) YZ plane—Simulated (blue dashed line) and Measured (red line) data in dB.

**Figure 25:**
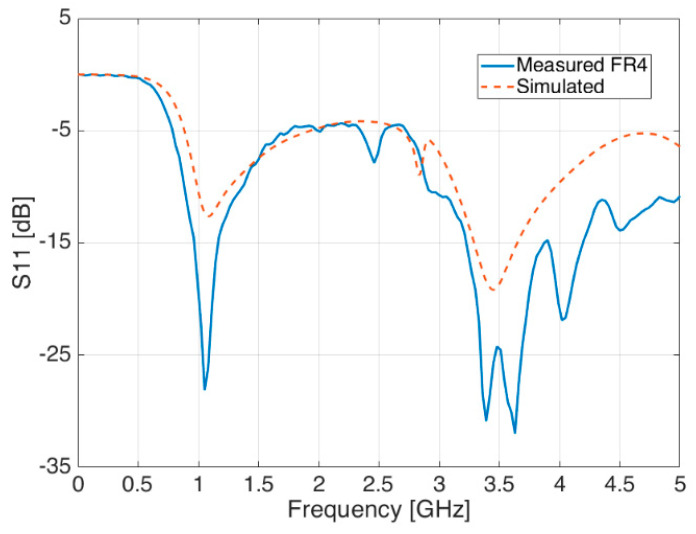
Reflection coefficient S_11_ of simulated antennas (red dashed line) and measured (blue continuous line) in FR-4 material.

Conversely to the aluminium case, this experimental setup was made of a signal generator Hittite HMC-T2100, a TDK Horn Tx reference antenna (1–18 GHz) and a spectrum analyser Agilent N1996A, in addition to the FR-4 blade Rx antenna under test. Despite the hand-made prototype and the absence of the CATR anechoic chamber, the agreement, both in terms of radiation patterns (Figure 23 and Figure 24) and reflection coefficient S_11_ (Figure 25) is still good, and also comparable in terms of shape and trends to the aluminium version introduced before.

In fact, as per the radiation diagrams of Figure 23 and Figure 24, it is still possible to see a fairly good resemblance with those of previous Figure 19 and Figure 20, measured in logarithm scale (dB) for the aluminium case.

It is worth noting that the little S_11_ peak around 2.8 GHz reported in Figure 25 among the simulations results (red dashed line) is also shown within the measurements (blue continuous line) but slightly shifted in frequency—about 400 MHz backwards. This peak seems to be a sign of a near resonance frequency related to some interactions between the blade antenna and the ‘C’ shape slot.

It is not yet possible to find an avionic antenna on the market with both ADS-B and 5G capabilities, but it is possible to find professional/commercial blade antennas that provide ADS-B only features with a similar design to the proposed one and that can, thus, become a first benchmark. Although their performances are not fully disclosed within their datasheets, the aluminum and FR-4 blade antenna series do provide overall good results both in terms of mechanical size and electrical properties. Actually, this proposed solution has a slightly smaller size in terms of height (shown in inches) and a better reflection coefficient (shown in VSWR) than the commercial ones, when benchmarked to [31,32] as an example, at no loss of performances even though the proposed solution accounts also for the 5G band—not currently addressed by any other avionic antenna so far.

## 5. Conclusions

Design of antennas for UAV applications over 5G networks definitely brings new challenges to designers when ADS-B capabilities are also added to the requirements. In this paper, a blade antenna at 1.09 GHz and 3.5 GHz for UAV applications was simulated and manufactured in two different materials. An incremental design was applied, in which the starting single band monopole performances were then compared to those of a dual band antenna, resulting in additional performances in terms of gain and impedance matching, thus, broadening the fields of application use. The proposed antenna is very simple to manufacture, and it requires low-cost materials, which makes it more appealing to the market. Matching characteristics of the blade antenna are evaluated under different bending conditions due to UAV installation on a fuselage. The same performances have been preliminary evaluated also in case of commercial drones, with no specific reported drawbacks. Results show matching performance acceptably robust in most cases, although a slight shift in the resonant frequency is seen. Overall, the performances of both the simulated antenna vs. the manufactured prototypes meet the desired requirements in terms of gain and—especially—return loss and radiation diagrams. Mechanical dimensions and weight constraints for practical applications have been fulfilled as well. Actually, when compared to commercial/professional blade antenna models for ADS-B communications, both aluminium and FR-4 series provided similarities in mechanical size and electrical performances, if not better ones.

Further investigations in this research project aim at higher 5G bands, i.e., 26 GHz or even 70 GHz frequency ranges. Lastly, to improve propagation efficiency on board of UAV, it would be wise to evaluate the optimum antenna placement on a UAV with regard to space diversity opportunities, as well as granting the antenna circular polarization for any challenging future application.

## Data Availability

Not applicable.
